# Additive roles of tobacco and cannabis co-use in relation to delay discounting in a sample of heavy drinkers

**DOI:** 10.1007/s00213-021-05993-7

**Published:** 2021-10-15

**Authors:** Steven J. Nieto, Alexandra Venegas, Elizabeth M. Burnette, James MacKillop, Lara A. Ray

**Affiliations:** 1 Department of Psychology, University of California Los Angeles, 1285 Franz Hall, Box 951563, Los Angeles, CA 90095-1563 USA; 2 Brain Research Institute, University of California Los Angeles, Los Angeles, CA USA; 3 Department of Psychiatry and Behavioural Neurosciences, McMaster University, Hamilton, ON Canada; 4 Department of Psychiatry and Biobehavioral Sciences, University of California, Los Angeles, Los Angeles, CA USA

**Keywords:** Delay discounting, Alcohol, Alcohol use disorder, Decision-making, Behavioral economics, Cannabis, Tobacco, Co-use, Polysubstance use

## Abstract

**Rationale:**

Alcohol use disorder (AUD) is associated with steeper delay discounting rates; however, it is unknown whether substance co-use, particularly cannabis use, has an additive effect on discounting rates among heavy drinkers. Furthermore, it is unclear whether substance co-use and delay discounting are independently associated with AUD severity.

**Objectives:**

The purpose of this study was to determine whether alcohol, tobacco, and cannabis co-use impacts delay discounting rates. We also sought to determine whether substance co-use and delay discounting were associated with AUD symptom counts.

**Methods:**

The study sample was culled from several human laboratory studies and consisted of 483 heavy drinking individuals who completed a baseline visit (prior to experimental procedures). Participants were divided into groups based on self-reported alcohol, tobacco, and cannabis use during the past 30 days: alcohol only (*n* = 184), alcohol + cigarettes (*n* = 89), alcohol + cannabis (*n* = 82), and tri-use (*n* = 128). We examined discounting rates across the 4 groups and used multiple linear regression to test whether co-use and delay discounting were associated with AUD symptoms.

**Results:**

After adjusting for covariates, individuals in the alcohol + cannabis group and the tri-use group had steeper discounting rates relative to the alcohol-only group. In addition, tri-use and delay discounting rates were independently correlated with a greater number of AUD symptoms.

**Conclusions:**

Delay discounting rates were significantly greater among subgroups reporting cannabis use providing partial support for an additive effect, while also highlighting the importance of co-use substance type. Both tri-use and delay discounting were associated with greater AUD severity, which may provide relevant intervention targets.

## Introduction

Substance co-use is common with ~ 80% of substance users regularly using more than one substance (Batel et al. 1995; Kalman et al. 2005). In the case of alcohol, co-use most often includes tobacco and cannabis (SAMHSA 2015). Specifically, 20–25% of current smokers are considered heavy drinkers (Dawson 2000; Spillane et al. 2020), and 20–50% of those with problematic alcohol use report using cannabis (Petry 2001), with the use of one independently increasing the probability of co-use of the two remaining substances within the same day (Roche et al. 2019). Co-use of alcohol and tobacco is associated with adverse negative health consequences compared to those who use either drug alone, such as brain injury and cancer risk (Durazzo et al. 2007; Ebbert et al. 2005). In a similar vein, co-use of alcohol and cannabis can also have legal- (e.g., driving under the influence of both substances), social-, and health-related consequences (Subbaraman and Kerr 2015; Terry-McElrath et al. 2014). Cannabis use is also associated with the development and maintenance of an AUD (Weinberger et al. 2016) and poor AUD treatment prognosis (Mojarrad et al. 2014; Subbaraman 2016). Despite the prevalence of simultaneous nicotine and cannabis use among heavy drinkers, whether co-use impacts decision-making and choice behavior is an understudied area.

Delay discounting, which refers to a greater preference for smaller immediate rewards compared to larger future rewards, is associated with a range of maladaptive behaviors, including addiction (Amlung et al. 2017; Bickel et al. 2019). In relation to problematic alcohol use, delay discounting is a proposed biomarker of AUD and its treatment (de Wit 2009; Kwako et al. 2018). Individuals with AUD exhibit greater discounting relative to healthy controls (Bobova et al. 2009; MacKillop et al. 2011, 2010). Furthermore, steeper discounting predicts binge-level alcohol consumption in social drinkers (Gowin et al. 2017) and greater motivated alcohol seeking in heavy drinkers during self-administration (Grodin et al. 2020). Thus, greater devaluation of future rewards may contribute to problematic alcohol use (Bickel et al. 2012b).

Steeper discounting of delayed rewards has been observed in substance users across several drug classes, including methamphetamine (Hoffman et al. 2006), cocaine (Coffey et al. 2003), nicotine (Bickel et al. 1999), and opioids (Kirby et al. 1999; Madden et al. 1997). Relative to the effect sizes observed in the aforementioned drug classes, the relationship between delay discounting and cannabis use is unclear (Strickland et al. 2020). While there is evidence that delay discounting is associated with cannabis use frequency and severity (Aston et al. 2016; Kim-Spoon et al. 2019; Lopez-Vergara et al. 2019; Sofis et al. 2020), there have also been null findings (Dennhardt et al. 2015; Heinz et al. 2013; Johnson et al. 2010; Peters et al. 2013). A recent meta-analysis found that the relationship between delay discounting and cannabis did not differ from other substances (Amlung et al. 2017). Whether the relationship between delay discounting and cannabis use is clinically meaningful remains unclear (Patel et al. 2020).

The co-use of multiple substances may further escalate the devaluation of future rewards beyond single-substance use. There are two opposing theories that address this relationship (Moody et al. 2016). The first hypothesizes that co-use use is associated with additive effects, such that each additional substance used increases the rate of discounting. The second hypothesizes that a ceiling effect may prevent further increments in discounting beyond that seen in mono-substance use alone. That is, heavy use of a single substance or alcohol is enough to induce devaluation of future rewards, and co-use of other substances does not have additive effects on discounting.

To date, the few studies that have examined the influence of co-use use on delay discounting have provided partial support for both hypotheses. Heavy drinking cigarette smokers displayed steeper delay discounting of small rewards relative to smokers only and heavy drinkers only (Moallem and Ray 2012). Cigarette smokers with another substance dependency discounted at a greater rate than smoking alone (Moody et al. 2016). Similarly, tri-substance users, who were alcohol-dependent, cocaine-dependent, and heavy smokers, discounted significantly more than heavy smokers only; however, delay discounting did not differ between tri-use and dual-use groups (Moody et al. 2016). There is also support for a ceiling effect. Cigarette smokers with and without substance dependence discounted at similar rates compared to either smoking alone or substance dependence alone (Businelle et al. 2010). Interestingly, delay discounting may also differ on the type of substance used and not the number of substance use disorders. For example, individuals with both cocaine and nicotine dependence, and cocaine dependence alone, had greater discounting compared to the nicotine-dependent group and control group (García-Rodríguez et al. 2013). Importantly, the cocaine-dependent group did not differ from the cocaine and nicotine-dependent group. Given these findings, it is possible that the use of certain substances results in steeper discounting compared to other substances.

Both co-use and greater delay discounting are associated with more severe alcohol use problems. Whether these relationships are independent of one another remains unknown. That is, it is presently unclear whether delay discounting is independently associated with AUD severity after adjusting for co-use. Additionally, the inclusion of cannabis users to address this research question is critically lacking. This is especially relevant considering many states in the US have legalized or decriminalized recreational cannabis use. Thus, in order to improve our understanding of the roles of co-use and delay discounting, the purpose of this study is to compare rates of delay discounting among heavy drinkers who self-report using alcohol only, alcohol + cigarettes, alcohol + cannabis, and tri-use over the last 30 days. An additional study goal is to identify whether co-use and delay discounting are independently associated with the clinical severity of AUD. Based on previous work, we hypothesize that co-users and tri-users will have steeper discounting relative to the alcohol-only group. We also postulate that co-use and delay discounting will be independently associated with AUD severity.

## Methods

### Participants

The current sample is culled from three separate clinical and experimental psychopharmacology studies with similar inclusion criteria and recruitment methods, all conducted in the Addictions Laboratory at the University of California, Los Angeles. Specifically, the samples analyzed herein were drawn from studies examining acute subjective responses to alcohol and alcohol self-administration (Bujarski et al. 2018), and naltrexone (Ray et al. 2018), and ibudilast (Ray et al. 2017) as pharmacotherapies for AUD. Although some studies involved pharmacological manipulations, all data analyzed herein were collected at a baseline assessment visit (i.e., prior to medication randomization or any experimental procedures). All studies recruited community samples of nontreatment-seeking drinkers from the Greater Los Angeles Area. All available discounting data across the three studies were utilized in the current study*.* All study procedures were approved by the University of California, Los Angeles Institutional Review Board, and all participants provided written informed consent after receiving a full explanation of the study procedures.

Interested individuals called the laboratory and completed a phone interview for preliminary eligibility. Heavy drinking was verified through one of the following methods: (i) greater than 7 drinks per week for females and greater than 14 drinks per week for males; (ii) an Alcohol Use Disorder Identification Test (AUDIT; (Saunders et al. 1993)) score of 8 or higher.

All studies had the following exclusion criteria: (i) current involvement in treatment programs for alcohol use or treatment engagement in the month prior to study participation (i.e., participants must not have engaged in treatment in the previous 30 days); (ii) use of nonprescription psychoactive drugs or use of prescription medications for recreational purposes; (iii) self-reported history of major mental illness (i.e., bipolar disorder or psychotic disorders); (iv) current use of antidepressants, mood stabilizers, sedatives, antianxiety medications, seizure medications, or prescription painkillers; (v) self-reported history of contraindicated medical conditions (e.g., chronic liver disease, cardiac disease); (vi) if female, pregnant (as verified by a urine sample), nursing, or planning to get pregnant in the next 6 months or refusal to use a reliable method of birth control; (vii) breath alcohol concentration (BAC) of greater than 0.000 g/dl as measured by the Dräger Inc. Alcotest® 6510; and (viii) positive urine toxicology screen for any drug (other than cannabis), as measured by Medimpex United Inc. 10 panel drug test.

### Measures

Across all studies, eligible participants were invited to the laboratory to complete a phenotypic battery consisting of sociodemographic (i.e., age, sex, education, income) and clinical measures.

Alcohol, cigarette, and cannabis use and problems were assessed using (a) the Timeline Followback (Sobell and Sobell 1992), an interview-based assessment of alcohol, cigarette, and cannabis use over the previous 30 days; (b) AUDIT (Saunders et al. 1993), an indicator of harmful and hazardous alcohol drinking; (c) The Fagerström Test for Nicotine Dependence (Heatherton et al. 1991) to assess dependence on nicotine; (d) Alcohol Dependence Scale (ADS) (Skinner et al. 1984), provides an index of alcohol dependence severity; (e) Cannabis Use Disorder Identification Test (Adamson and Sellman 2003) to screen for harmful and hazardous cannabis use; (f) The Structured Clinical Interview of DSM-5 (SCID), administered by a master’s level clinician to assess for current AUD symptoms.

Delay discounting was assessed using the Monetary Choice Questionnaire (MCQ) (Kirby et al. 1999), a well-validated delay discounting measure. The measure consists of 27 dichotomous choices between smaller-immediate and larger-delayed monetary rewards that are preconfigured to provide estimates of an individual’s delay discounting rate. In this study, reward amounts were hypothetical and not tied to participant compensation. Individual responses on the MCQ were processed using a freely available, automated tool (Kaplan et al. 2016). Participants with consistency values less than 75% (*n* = 4) were excluded from statistical analyses as such scores may reflect low attention/effort on the questionnaire.

### Data analytic approach

The delay discounting task has a unique scoring system as it is not consistent over time, but rather a hyperbola-like function so that the reward disproportionately gains value as the time to receipt approaches and disproportionately loses value when initially delayed. The hyperbolic function is characterized by the equation *Vd* = *V*/(1 + *kd*) in which *Vd* is the present discounted value of the reward, *V* is the objective value of the reward, *k* is a constant that reflects the rate of discounting, and *d* is the temporal delay. Therefore, a higher *k* value indicates a more impulsive tendency to prefer smaller, immediate rewards over larger, future rewards. As *k* is not normally distributed, we use ln(*k*) as the interpretable delay discounting score.

Co-use groups were classified according to their self-reported use on the Timeline Followback as follows: (1) alcohol only, (2) alcohol + cigarettes, (3) alcohol + cannabis, and (4) tri-use (alcohol + cigarettes + cannabis). Although cigarette users may have used other tobacco products, this information was not collected. A series of one-way analyses of covariance (ANCOVA) was used to compare co-use groups on continuous demographic and clinical measures. Cochran–Mantel–Haenszel (CMH) tests, an extension of chi-square tests allowing for covariates, were used to compare groups on categorical measures. Statistical significance was set at *p* < 0.025 at the omnibus test level for these analyses. Statistical significance was set at *p* < 0.05 for the additional analyses. A one-way ANCOVA was used to identify whether co-use groups differed on delay discounting while adjusting for sociodemographic covariates and AUD symptom counts. To further control type 1 error rate, Tukey post hoc tests were used to follow up significant ANCOVA omnibus tests. Multiple linear regression was used to determine whether co-use use and delay discounting were associated with AUD symptom count (dimensional outcome variable) while adjusting for covariates. In addition to controlling for sociodemographic covariates, the study source was used as a three-level categorical covariate in all analyses. Statistical analyses were conducted using PROC GLM in SAS 9.4 software (Cary, NC, USA).

## Results

### Sample characteristics and differences

Participants were classified into one of four use groups: alcohol only (*n* = 184, 38.09%), alcohol + cigarettes (*n* = 89, 18.43%), alcohol + cannabis (*n* = 82, 16.98%), and tri-use (*n* = 128, 26.50%). Sample and clinical characteristics are presented in Table 1. Tukey post hoc tests showed that the alcohol + cannabis group was younger compared to the alcohol-only and alcohol + cigarettes groups. The tri-use group was younger relative to the alcohol + cigarettes group. The alcohol-only group had fewer drinks per drinking day compared to the alcohol + cigarettes and tri-use groups. The alcohol + cannabis group had fewer drinks per drinking day compared to the tri-use group. The tri-use group had higher AUDIT scores and a greater number of DSM-5 AUD symptoms than the alcohol-only and alcohol + cannabis groups. Given the group differences in age, sex, and AUD symptoms, these measures were included as covariates in subsequent analyses where appropriate.

**Table 1:** Demographic and clinical characteristics by alcohol and substance use groups controlling for study source

Means (SD) or *n* (%)							
	Alcohol only (***n*** = 184)	Alcohol + cigarettes (***n*** = 89)	Alcohol + cannabis (***n*** = 82)	Tri-use (***n*** = 128)	Statistic	***p***	Effect size
Age^b,d,e^	29.65 ± 8.13	31.62 ± 8.25	26.91 ± 5.04	28.78 ± 7.79	*F* = 7.08	*p* < 0.0001	$${{{\eta}}}_{{{p}}}^{2}$$= 0.045
Sex (male)	102 (54.35%)	61 (68.54%)	53 (64.63%)	97 (75.78%)	*CMH *= 16.59	*p* = 0.0009	–
Education					*CMH* = 29.73	*p* = 0.053	–
Less than high school	1 (0.53%)	1 (1.12%)	2 (2.44%)	18 (14.06)			
High school/GED	52 (28.26%)	34 (38.20%)	21 (25.61%)	54 (42.19%)			
2-year college	24 (13.37%)	14 (15.73%)	10 (12.20%)	23 (17.97%)			
4-year college	85 (45.45%)	31 (34.83%)	43 (52.44%)	27 (21.09%)			
Masters	17 (9.09%)	8 (8.89%)	6 (7.32%)	6 (4.69%)			
Doctoral	2 (1.07%)	0 (0.00%)	0 (0.00%)	0 (0.00%)			
Professional	3 (1.60%)	1 (1.12%)	0 (0.00%)	0 (0.00%)			
Income					*CMH* = 8.05	*p* = 0.235	**–**
Below $30,000	74 (40.22%)	38 (42.70%)	42 (51.22%)	59 (55.14%)			
$30,000–$74,999	67 (36.41%)	30 (33.71%)	22 (26.83%)	35 (32.71%)			
Above $75,000	43 (23.37%)	21 (23.60%)	18 (21.95%)	13 (12.15%)			
Drinking days	14.55 ± 7.74	16.11 ± 7.64	14.33 ± 6.64	17.71 ± 7.43	*F* = 2.77	*p* = 0.041	$${\eta }_{p}^{2}$$= 0.017
Drinks per drinking day^a,c,f^	4.82 ± 2.85	5.91 ± 3.25	5.11 ± 2.50	6.43 ± 3.43	*F* = 7.04	*p* = 0.0001	$${\eta }_{p}^{2}$$= 0.043
AUDIT^c, f^	14.23 ± 6.85	15.60 ± 7.15	13.59 ± 5.16	17.80 ± 8.03	*F* = 5.80	*p* = 0.0007	$${\eta }_{p}^{2}$$= 0.035
ADS^c^	11.22 ± 6.71	11.83 ± 6.69	11.32 ± 5.46	14.00 ± 7.36	F = 3.45	*p* = 0.017	$${\eta }_{p}^{2}$$= 0.022
AUD symptom count^c,f^	2.32 ± 2.45	2.94 ± 2.38	2.13 ± 1.99	3.32 ± 2.63	*F* = 4.72	*p* = 0.003	$${\eta }_{p}^{2}$$= 0.030
Cigarette smoking days	N/A	18.09 ± 11.75	N/A	17.36 ± 11.74	*F* = 0.65	*p* = 0.422	$${\eta }_{p}^{2}$$= 0.002
FTND	N/A	3.13 ± 1.43	N/A	2.62 ± 1.67	*F* = 3.22	*p* = 0.074	$${\eta }_{p}^{2}$$= 0.009
Cannabis use days	N/A	N/A	9.30 ± 10.45	9.21 ± 9.11	*F* = 0.11	*p* = 0.743	$${\eta }_{p}^{2}$$= 0.0005
CUDIT	N/A	N/A	8.19 ± 6.75	8.71 ± 6.00	*F* = 0.25	*p* = 0.616	$${\eta }_{p}^{2}$$= 0.001

*BDI-II*, Beck Depression Inventory-II; *BAI*, Beck Anxiety Inventory; *AUDIT*, Alcohol Use Disorders Identification Test; *ADS*, Alcohol Dependence Scale; *PACS*, Penn Alcohol Craving Scale; *AUD*, alcohol use disorder; *DrInC*, Drinker Inventory of Consequences; *FTND*, Fagerström Test for Nicotine Dependence; *CUDIT*, Cannabis Use Disorder Identification Test; *CMH*, Cochran–Mantel–Haenszel general association statistic; statistical significance for the omnibus test was set at *p* < 0.025.

^a^Alcohol-only and alcohol + cigarettes groups differ, *p* < 0.05.

^b^Alcohol-only and alcohol + cannabis groups differ, *p* < 0.05.

^c^Alcohol-only and tri-use groups differ, *p* < 0.05.

^d^Alcohol + cigarettes and alcohol + cannabis groups differ, *p* < 0.05.

^e^Alcohol + cigarettes and tri-use groups differ, *p* < 0.05.

^f^Alcohol + cannabis and tri-use groups differ, *p* < 0.05.

### Co-use and delay discounting

**Fig. 1:**
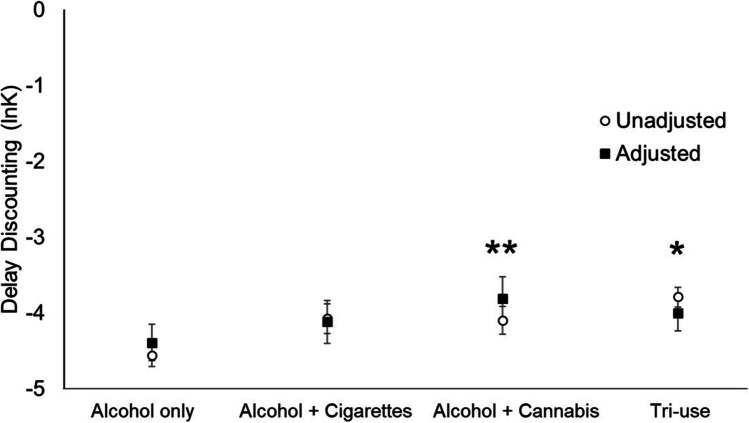
Co-use profiles and delay discounting. Open circles indicate unadjusted group means $\pm$ SEM, and closed squares indicate adjusted group means $\pm$ SEM. *p < 0.05. **p < 0.01

A one-way ANCOVA adjusting for study source, age, sex, education, income, and AUD symptom count revealed a main effect of co-use on delay discounting, *F* (3, 461) = 2.49, *p* = 0.042; ${\eta }_{p}^{2}$ = 0.02. Unadjusted and adjusted group means are shown in Fig. 1. Age (*p* < 0.0001; ${\eta }_{p}^{2}$ = 0.05), education (*p* = 0.011; ${\eta }_{p}^{2}$ = 0.04), income (*p* = 0.031; ${\eta }_{p}^{2}$ = 0.02) and AUD symptom count (*p* = 0.018; ${\eta }_{p}^{2}$ = 0.01) were statistically significant covariates. Study source and sex were not significantly associated with delay discounting (*p*’s > 0.05). Tukey post hoc tests showed that among use groups, the alcohol + cannabis group (*p* = 0.008) and tri-use group (*p* = 0.043) had steeper discounting rates compared to the alcohol-only group.

### AUD symptom count, co-use, and delay discounting

Multiple linear regression was used to identify whether co-use and delay discounting were independently associated with AUD symptom count while also adjusting for covariates (Table 2). The linear regression model explained 18.24% of the variability in AUD symptom count. Tri-use (*B* = 0.649; *p* = 0.030) was positively associated with AUD symptom count controlling for sociodemographic covariates and delay discounting. Greater delay discounting (*B* = 0.147; *p* = 0.025) was significantly associated with AUD symptom count while adjusting for sociodemographic covariates and co-use. Older age (*B* = 0.015; *p* = 0.016) was significantly associated with AUD symptom count, but sex, education, and income were not correlated with AUD symptom count (*p*’s > 0.05).

**Table 2:** Linear regression model of AUD symptom count

Variable	*B*	SE	95% C.I	*p*
Age	0.015	0.015	0.006–0.066	0.016
Sex^a^	0.298	0.234	− 0.063–0.863	0.203
Education^b^				
High school/GED	− 0.840	0.827	− 2.486–0.640	0.310
2-year college	− 0.621	0.852	− 2.437–0.787	0.465
4-year college	− 0.898	0.837	− 2.705–0.452	0.283
Masters	− 1.077	0.907	− 3.061–0.378	0.236
Doctoral	− 0.268	1.801	− 3.920–3.201	0.881
Professional	− 1.084	1.404	− 4.082–1.407	0.441
Income^c^				
$30,000–$74,999	0.206	0.243	− 0.365–0.603	0.399
Above $75,000	− 0.158	0.292	− 0.848–0.313	0.590
Co-use group^d^				
Alcohol + cigarettes	0.488	0.297	− 0.124–1.053	0.101
Alcohol + cannabis	− 0.169	0.307	− 0.740–0.474	0.582
Tri-use	0.649	0.288	0.060–1.204	0.030
Delay discounting (lnk)	0.147	0.065	0.024–0.296	

^a^Reference category is "female”.

^b^Reference category is “less than high school education”.

^c^Reference category is “below $30,000”.

^d^Reference category is “alcohol-only group”.

## Discussion

The current study sought to evaluate the role of delay discounting among alcohol, cigarette, and cannabis co-users. In line with previous work, we found that tri-users had more drinks per drinking day, greater alcohol dependence severity, and more alcohol-related consequences relative to the alcohol-only and alcohol + cannabis groups. Tri-users did not significantly differ from the alcohol + cigarette group on any alcohol measure. Given the well-established negative impact of simultaneous alcohol and cigarette use, it is likely that cigarette co-use is driving the effect on alcohol dependence severity. That is, once heavy drinkers use cigarettes regularly, the additive effect of cannabis use on alcohol use severity may be minimal.

In partial support of our hypothesis, the alcohol + cannabis group and tri-use group had steeper rates of delay discounting relative to the alcohol-only group. Steeper discounting rates in the alcohol + cannabis group were surprising given that cigarette and alcohol co-use appeared to drive alcohol dependence severity. However, the impact of cannabis on delay discounting is an understudied research domain, and these results await replication. Delay discounting is hypothesized to be a transdiagnostic process (Amlung et al. 2019; Lempert et al. 2019) and a dimension of executive function (Bickel et al. 2012a). Thus, steeper discounting in the cannabis use groups may be the result of cognitive deficits (e.g., memory) as a result of cannabis use (Volkow et al. 2016). However, the literature in this domain is inconsistent as some studies fail to find associations between cannabis use and delay discounting (Johnson et al. 2010; Petker et al. 2019; Strickland et al. 2017). We did not find an additive effect of alcohol and cigarette use compared to mono-use of either substance alone, which supports (Businelle et al. 2010) and contrasts (Moallem and Ray 2012; Moody et al. 2016) previous work. Cannabis and other drug use, either reported and not tested or unknown, in these previous studies may add heterogeneity in discounting rates. This is especially noteworthy as certain substances can have a selective and greater impact on delay discounting compared to the number of substances used (García-Rodríguez et al. 2013).

Our current findings are in partial support of an additive effect; however, this effect was only observed in heavy drinkers who engaged in cannabis use or tri-use. Our sample was composed of heavy drinkers, most of whom (60%) had an AUD, from across several human laboratory studies conducted in our laboratory. Very few of the individuals in the current study met DSM-5 criteria for another substance use disorder (*n* = 8). As a result, it is important to clarify that while the level of co-use of cigarettes and cannabis was similar across groups, the average level of co-use did not meet DSM-5 criteria for a substance use disorder and does not speak to comorbidity between multiple AUD/SUDs. However, our findings do speak to the large numbers of individuals who are concurrent recreational users and those who use at subclinical levels. Future work among individuals with polysubstance use disorder may further elaborate on the connection between co-use and delay discounting from the perspective of diagnostic comorbidity. Nevertheless, the co-use perspective remains highly relevant as it includes a broader segment of the population and is associated with a host of health and clinical consequences. It is important to note that well-established risk factors associated with AUD and polysubstance use, such as sex, family history of alcohol problems, and trait impulsivity may have impacted the findings of the current study. While our statistical models adjusted for sociodemographic variables (i.e., sex, income, education), our study cannot rule out other contributing factors that might predispose individuals to polysubstance use.

Delay discounting and tri-use both were independently associated with AUD severity via increases in AUD symptom count. Symptom count is a plausible outcome given that all participants engaged in heavy drinking and that we sought to establish the clinical significance of the co-use variables and delay discounting, tested simultaneously. While co-use might impact delay discounting rates, even after adjusting for other variables in the model, both tri-use and delay discounting explain a unique amount of variance in AUD severity. This finding is in line with previous findings that cannabis and nicotine co-use is associated with heavier alcohol use compared to either substance alone (Agrawal et al. 2012; Peters et al. 2013; Ramo et al. 2012; Schauer and Peters 2018).

Previous work has shown that steeper discounting rates are primarily driven by AUD and not comorbid psychopathology. While discounting is greater in individuals with current AUD compared to healthy controls and individuals with past AUD, there is no additive effect of psychopathology (including cannabis and nicotine dependence)(Gowin et al. 2019). While our work does not address the question of additive effects of comorbid psychopathology on AUD severity directly, we do observe that after adjusting for differences in discounting rates, tri-use was associated with more AUD symptoms compared to alcohol only.

Both substance co-use and delay discounting may serve as intervention targets. Several behavioral and pharmacological treatments have been developed for individuals who engage in substance co-use. For example, the nicotinic acetylcholine receptor agonist varenicline is effective in reducing both cigarette and alcohol use behaviors (Falk et al. 2015; McKee and Weinberger 2013; Mitchell et al. 2012). Behavioral economic interventions, such as the community reinforcement approach (Hunt and Azrin 1973) and contingency management (Stitzer and Petry 2006), are effective for addressing alcohol problems by focusing on altering reinforcement contingencies in the individual’s life to increase the value of abstinence. Additionally, working memory training decreased discounting rates in stimulant users (Bickel et al. 2011) and alcohol consumption in problem drinkers (Houben et al. 2011). Although behavioral economic interventions may offer effective treatment options, randomized clinical trials including these interventions, even as adjunctive treatments, are critically lacking. While the implication of these findings to clinical practice remains speculative, it is noteworthy that delay discounting is actively under study as a treatment target for addiction (ClinicalTrials.gov Identifier: NCT04139148; NCT04449055).

The results from this study should be interpreted in relation to its strength and limitations. Strengths of the study include large sample size, use of well-validated measures, and well-powered analyses. Limitations include the possibility of recall bias on self-report measures, as well as the lack of behavioral measures of delay discounting and real rewards. The latter concern is partially mitigated by findings that hypothetical discounting rates are strongly associated with discounting of real rewards (Lagorio and Madden 2005). Additionally, we are unable to provide fine-grained measurements of simultaneous use of alcohol and cannabis/cigarettes among our sample. Simultaneous use of alcohol and substances, particularly cannabis, in the same episode can result in heavier alcohol use (Terry-McElrath et al. 2013), more alcohol-related consequences (Brière et al. 2011), and AUD (Midanik et al. 2007) relative to those who use both substances on separate occasions. It is possible that individuals who engage in simultaneous use might have greater discounting rates, and that discounting rate may serve as a mediator between simultaneous co-use and AUD severity. Thus, examining the relationship between simultaneous use and delay discounting may be a promising area for future work.

In summary, we identified cigarette and cannabis co-use profiles, which differ on clinical measures of alcohol use. We observe that both the alcohol + cannabis group and tri-use have steeper discounting rates compared to alcohol-only and alcohol + cigarette groups. In addition, tri-use and delay discounting were associated with greater AUD severity compared to the alcohol-only group. Thus, simultaneous alcohol, cigarette, and cannabis use, as well as delay discounting, represent independent risk factors for more severe AUD, such that both of these clinical features should be considered in clinical settings.
